# Molecular Characteristics and Serodiagnostic Potential of Dihydrofolate Reductase from *Echinococcus granulosus*

**DOI:** 10.1038/s41598-017-00643-5

**Published:** 2017-03-31

**Authors:** Xingju Song, Dandan Hu, Min Yan, Yu Wang, Ning Wang, Xiaobin Gu, Guangyou Yang

**Affiliations:** Department of Parasitology, College of Veterinary Medicine, Sichuan Agricultural University, Chengdu, China

## Abstract

The larval stage of *Echinococcus granulosus* causes cystic echinococcosis (CE), a neglected tropical disease that leads to morbidity and mortality in humans and livestock worldwide. Here, we identified and characterized dihydrofolate reductase (Eg-DHFR) from *E. granulosus*, and evaluated its potential as a diagnostic antigen for sheep CE. Comparison between mammalian (host) DHFR and Eg-DHFR indicates that 45.7% of the 35 active site residues are different. Immunolocalisation analysis showed that native Eg-DHFR was widely distributed in all life-cycle stages of *E. granulosus*. Recombinant Eg-DHFR (rEg-DHFR) showed typical DHFR enzymatic parameters towards substrate, and was very sensitive to inhibition by methotrexate (IC_50_ = 27.75 ± 1.03 nM) and aminopterin (IC_50_ = 63.67 ± 6.76 nM). However, inhibition of DHFR exhibited little protoscolicidal effect *in vitro*. As there is no reliable method to monitor sheep CE, the immunogenicity of rEg-DHFR was detected, and we developed an indirect ELISA (iELISA) for CE serodiagnosis. The iELISA exhibited diagnostic specificity of 89.58%, diagnostic sensitivity of 95.83%, and the diagnostic accuracy was 91.67% compared with necropsy. Cross-reactivity assay showed analytical specificity of 85.7%. These suggest that rEg-DHFR is an effective antigen for the diagnosis of sheep CE.

## Introduction

Cystic echinococcosis (CE) is a cosmopolitan zoonosis, caused by the larval stage of *Echinococcus granulosus*, which affects humans and a wide range of mammalian intermediate hosts^1, 2^. It is mainly transmitted through definitive hosts (the canids) carrying the adult stage in the small intestine and releasing eggs along with their faeces; then, intermediate hosts ingest the eggs and develop larval stages (hydatid cysts). CE causes a large global burden, which was estimated to be 1–3.6 million disability-adjusted life years lost annually for human CE^3^ and $2 billion lost annually in the livestock industry^4^. The mortality rate of human CE is about 2–4%^5^. However, in many endemic regions, the incidence rates in humans can be over 50 per 100,000 person-years and the prevalence may reach 5–10%^6–8^.

There is a long history of attempting serology for CE in humans and attempts are still being made to optimise serological methods^9–11^. The most common method is ELISA using *E. granulosus* hydatid cyst fluid antigen (HCF)^5^. However, natural HCF is difficult and expensive to prepare, and cannot be produced commercially. Specific recombinant antigens have good potential as diagnostic and follow-up tools for CE, but progress in this field is hampered by a lack of standardisation^12, 13^. Compared with human cases, natural infections in sheep produce relatively poor antibody responses^14, 15^. This is thought to be the result of antigen sequestration, rather than immunological tolerance or non-specific immunosuppression^16^. Therefore, an inexpensive, accurate immunodiagnostic assay as a monitoring tool for diagnosis of *E. granulosus* infection in sheep is necessary^17^. However, relatively little research has been undertaken on development of immunodiagnostic techniques for CE in domesticated animals such as sheep and cattle^18–20^. These assays use natural *E. granulosus* hydatid cyst fluid antigens, which is difficult to purify and standardize. Meanwhile, the use of recombinant components in the diagnosis of sheep CE shows low sensitivities in some cases^15, 21^. Thus, screening for a new antigenic component with high diagnostic sensitivity and specificity is a crucial task for improving the diagnosis of sheep CE^22^.

Dihydrofolate reductases (DHFRs) are ubiquitous enzymes. They catalyse the NADPH-dependent reduction of 7, 8-dihydrofolate (DHF) to 5, 6, 7, 8-tetrahydrofolate, a vital co-factor for purine and thymidylate synthesis in cells^23, 24^. Extensive research has been directed towards the development of DHFR inhibitors as a therapeutic target for the treatment of parasitic protozoan infections^25, 26^. DHFRs have been highlighted as a potential insecticide target based on differences in active site residues between the insect enzyme and vertebrate DHFR^27^. Thus, the structural requirements for inhibitors have been studied extensively and novel agents that utilise the different active sites of the host and parasitic enzymes have been proposed^28^.

Given that no information on tapeworm DHFR is available, the aims of the present study were (i) to analyse the enzymatic activity characteristics of DHFR from *E. granulosus* (Eg-DHFR); (ii) to assess the potential of Eg-DHFR as a target for *E. granulosus* control chemicals; (iii) to express recombinant Eg-DHFR (rEg-DHFR) and locate the native protein in different stages of the parasite; and (iv) to detect the immunogenicity of rEg-DHFR and develop an indirect enzyme-linked immunosorbent assay (ELISA) for diagnosis of CE in sheep.

## Results

### Sequence analysis of Eg-DHFR

**Figure 1:**
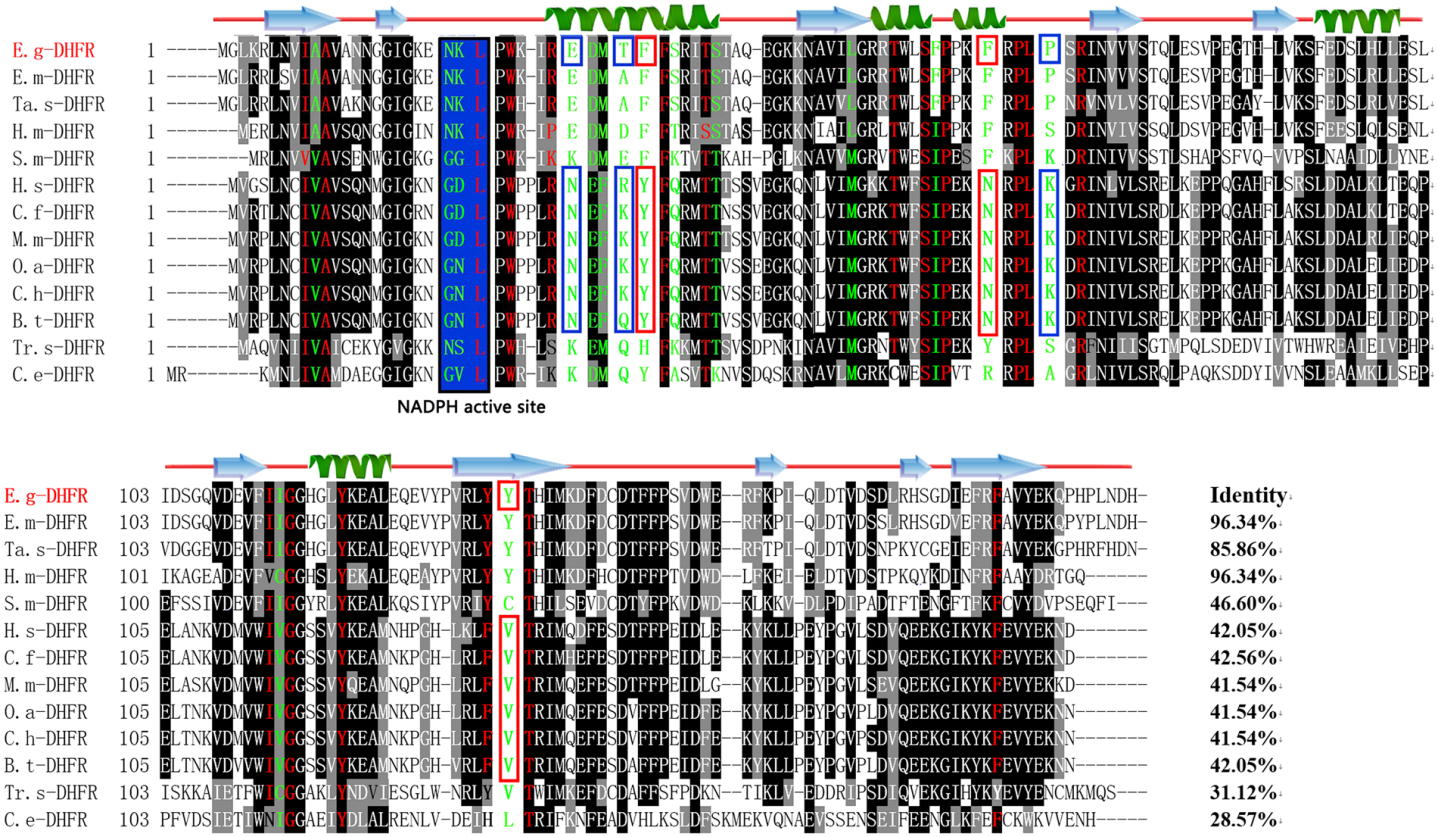
Sequence alignment analysis of *E. granulosus* DHFR. Alignment of the deduced amino acid sequence of Eg-DHFR (GeneDB: EgrG_000572400) with homologues from other species. The percentage homology of Eg-DHFR with each DHFR is shown at the end of the alignment. Different active-site residues between mammalian (host) and *E. granulosus* DHFRs are indicated by green letters, and identical active-site residues are indicated by red letters. Active-site residues with charge changes are marked with blue boxes (mammalian host - *E. granulosus*, Asn30 - Glu30, Asp/Asn22 - Lys22, Arg/Lys33 - Thr33, and Lys68 - Pro68). The exchange of polar and nonpolar residues (Tyr34 - Phe34, Asn64 - Phe64, Val136 - Tyr135) is marked with red boxes. The NADPH binding-site is highlighted with a blue background. The predicted secondary structure of Eg-DHFR is displayed above the alignment. Accession numbers: *Echinococcus granulosus* DHFR (E.g-DHFR) GeneDB: EgrG_000572400; *Echinococcus multilocularis* DHFR (E.m-DHFR) GenBank: CDS38333.1; *Taenia solium* DHFR (Ta.s-DHFR) GenBank: PEL761437; *Hymenolepis microstoma* (H.m-DHFR) GenBank: CDS28294.1; *Schistosoma mansoni* DHFR (S.m-DHFR) GenBank: XM_002580511; *Homo sapiens* DHFR (H.s-DHFR) GenBank: AC130896; *Canis familiaris* DHFR (C.f-DHFR) GenBank: 003432480; *Mus musculus* DHFR (M.m-DHFR) GenBank: NP_034179; *Ovis aries* DHFR (O.a-DHFR) GenBank: GO728717; *Capra hircus* DHFR (C.h-DHFR) GenBank: JO612108; *Bos taurus* DHFR (B.t-DHFR) GenBank: NM_001077883; *Trichinella spiralis* DHFR (Tr.s-DHFR) GenBank: ES273161; *Caenorhabditis elegans* DHFR (C.e-DHFR) GenBank: CAB02272.1.

**Figure 2:**
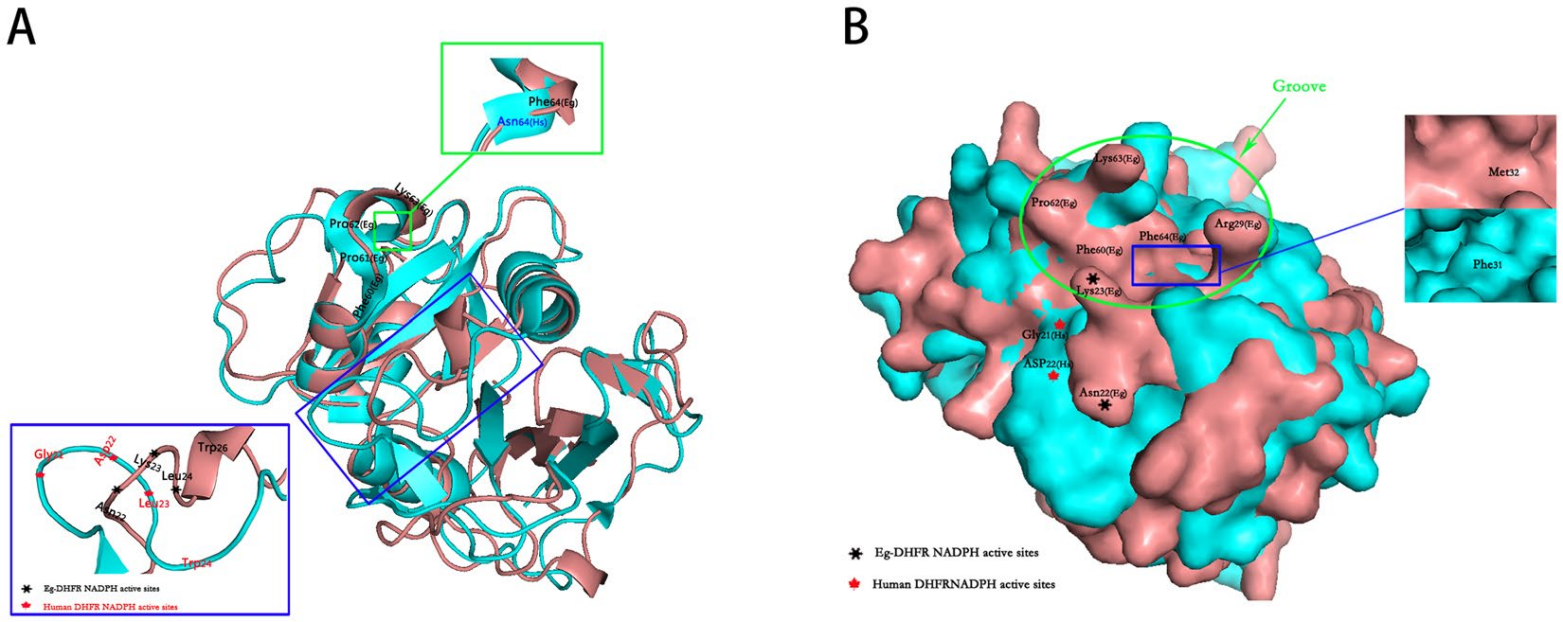
Comparison of the three-dimensional structure of DHFR between *E. granulosus* and *Homo sapiens*. The 3D structure of Eg-DHFR was modelled based on the crystal structure of *Schistosoma mansoni* DHFR (PDB accession code: 3vco.1). The global and per-residue model quality of Eg-DHFR has been assessed using the QMEAN scoring function (GMQE and QMEAN value were 0.76 and −0.14, respectively). 3D structure of Eg-DHFR (deepsalmon) was aligned to DHFR of *H. sapiens* (cyan; PDB accession code: 3gyf.1), and shown as cartoon (**A**) and surface (**B**). Important structural difference were enlarged.

The cDNA encoding Eg-DHFR contained an open reading frame of 576 bp, corresponding to a protein of 191 amino acid residues. The predicted molecular mass of the protein was 21.9 kDa. Multiple sequence alignment revealed that Eg-DHFR had high similarity with the DHFRs from *E. multilocularis* (96.34%), *Hymenolepis microstoma* (96.34%) and *Taenia solium* (85.86%), and shared 41.54–42.56% overall identity with DHFRs from mammalian hosts (Fig. 1). Previous studies on mammalian DHFRs showed there are 35 active site residues in the enzyme^29^. Comparison of the active site residues between mammalian DHFRs and Eg-DHFR indicated that there are 19 conserved residues and 16 different residues (i.e., 45.7% of the total active site residues are different). Otherwise, it is worth to note the following points: (i) charge changes occurred between mammalian host and *E. granulosus* DHFR at four sites, including the loss of a negative charge (mammalian host - *E. granulosus*, Asn30 - Glu30), the addition of a positive charge (Asp/Asn22 - Lys23), and the loss of a positive charge (Arg/Lys33 - Thr33, Lys68 - Pro68); (ii) further changes were the exchange of polar and nonpolar amino acids (Tyr34 - Phe34, Asn64 - Phe64, Val136 - Tyr135) (Fig. 1). Although the modelled three-dimensional structure of Eg-DHFR is conserved compared with homologous DHFRs, conformation changes were predicted in some active site residues (e.g., *H. sapiens* - Eg-DHFR: Gly21 - Asn22, Asp22 - Lys23, Trp24 - Trp26, Phe31 - Met32, Asn64 - Phe64) (Fig. 2).

### Expression and identification of rEg-DHFR

**Figure 3:**
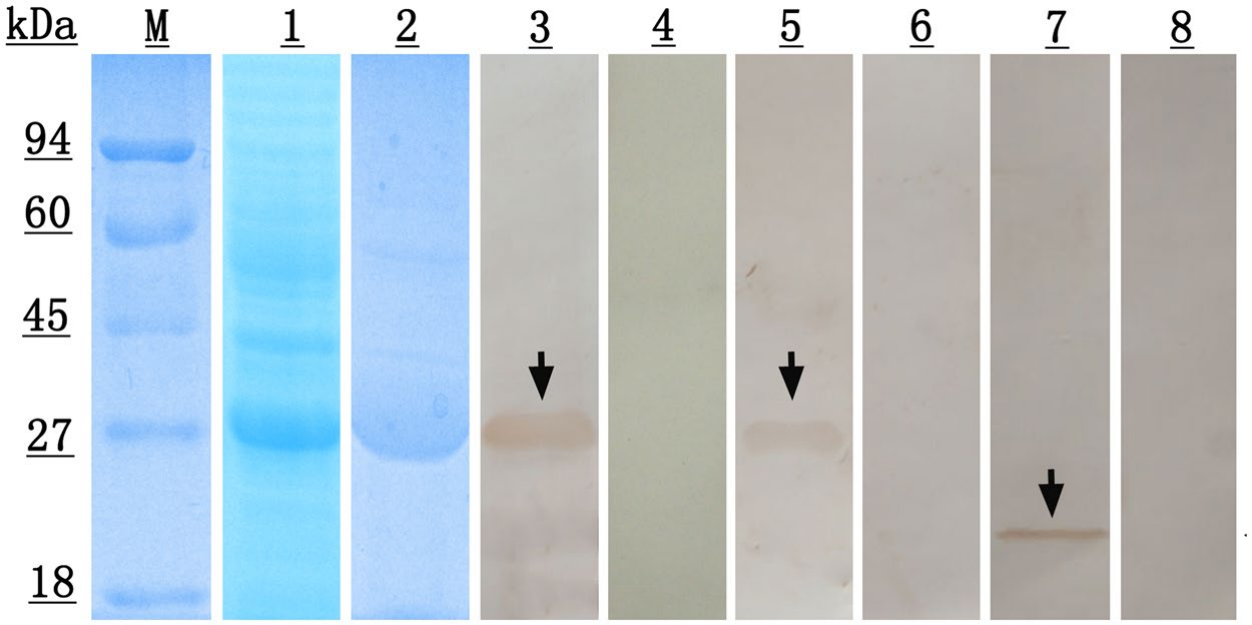
SDS-PAGE and western blotting analysis of Eg-DHFR. M, protein molecular weight markers; lane 1, *E. coli* BL21 (DE3) lysate from IPTG-induced cells expressing rEg-DHFR; lane 2, purified rEg-DHFR; lane 3, purified rEg-DHFR probed with anti-rEg-DHFR rabbit serum; lane 4, purified rEg-DHFR probed with native (preimmune) rabbit serum; lane 5, purified rEg-DHFR probed with the serum of *E. granulosus* infected sheep; lane 6, purified rEg-DHFR probed with native (healthy) sheep serum; lane 7, the total protein from protoscoleces probed with anti-Eg-DHFR rabbit serum; lane 8, the total protein from protoscoleces probed with native (preimmune) rabbit serum.

The Eg-DHFR gene was successfully inserted into the pET28a (+) expression vector and a soluble protein was expressed in *Escherichia coli* BL21 (DE3) cells following induction. The purified recombinant protein showed a single band in 12% SDS-PAGE, which was similar in size to the predicted ~26 kDa (including a His-tag). In western blotting analysis, a single ~26 kDa band was recognised using both rabbit anti-rEg-DHFR IgG (positive control) and sheep anti-*E. granulosus* serum (experimental group), which suggested strong reactivity and good antigenicity of this recombinant protein (Fig. 3). In addition, anti-rEg-DHFR rabbit IgG recognised a ~22 kDa protein from extracts of *E. granulosus* protoscoleces (PSCs), corresponding to the size of native Eg-DHFR. No band was recognised by native (preimmune/uninfected) serum from rabbit and sheep.

### Immunolocalisation of endogenous Eg-DHFR

**Figure 4:**
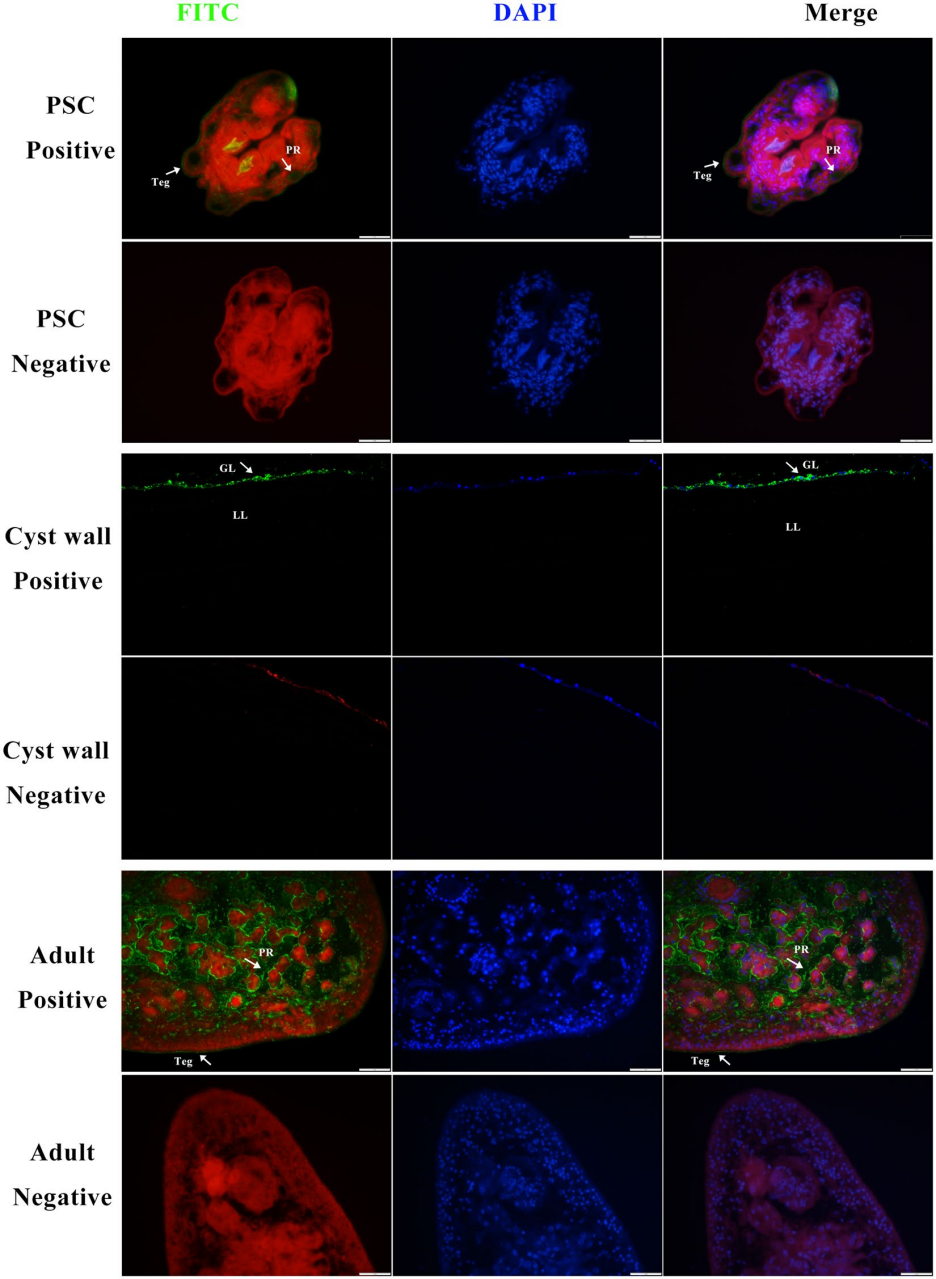
Immunofluorescent localisation of Eg-DHFR in different stages of *E. granulosus*. Eg-DHFR was localised in the protoscolex, germinal layer and adult worm using specific anti-rEg-DHFR IgG (positive), or preimmune serum (negative). The nucleus DNA was stained with DAPI (blue). Abbreviations: Teg, tegument; PR, parenchymal region; GL, germinal layer; LL, laminated layer. Scale bars: 1 mm.

Endogenous Eg-DHFR was localised in different life-cycle stages of *E. granulosus* by an immunofluorescence method using rabbit rEg-DHFR antibody. In PSCs, the fluorescence signals were mainly localised in the parenchymal region, while weak signals were also detected in the tegument tissues (Fig. 4). Fluorescent signals were observed in the germinal layer. In adult worms, Eg-DHFR was widely distributed in the parenchymal region and tegument tissue (Fig. 4), and especially distributed in the rostellum and suckers of the scolex (see Supplementary Fig. S1). No fluorescence signals were detected in the negative controls.

### Enzymatic activity of rEg-DHFR and inhibitory effect of antifolates

**Figure 5:**
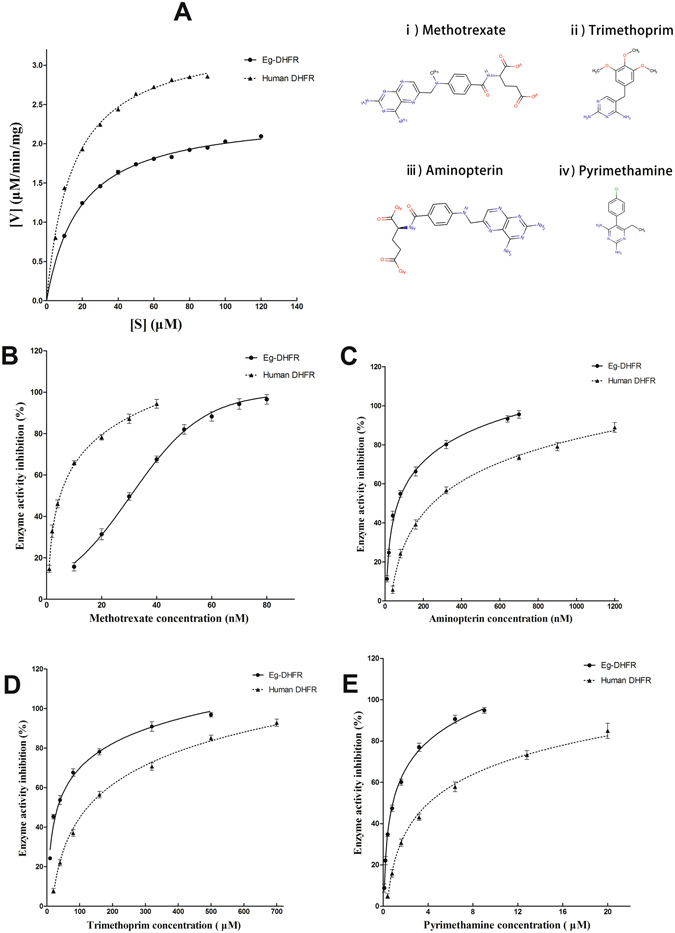
Enzymatic activity of rEg-DHFR and inhibitory effect of antifolates. (**A**) The kinetic parameters were calculated by using a Michaelis-Menten curve fit using GraphPad Prism® software (nonlinear regression). (**B**) Inhibition curves of rEgDHFR and human DHFR enzyme activity by methotrexate. (**C**) Inhibition curves of rEgDHFR and human DHFR enzyme activity by aminopterin. (**D**) Inhibition curves of rEgDHFR and human DHFR enzyme activity by trimethoprim. (**E**) Inhibition curves of rEgDHFR and human DHFR enzyme activity by pyrimethamine. The IC_50_ of antifolates were calculated and represented using means standard errors of the means. Chemical structures of the antifolate drugs methotrexate (i), trimethoprim (ii), aminopterin (iii), and pyrimethamine (iv).

The kinetic properties of rEg-DHFR and human DHFR were investigated. By using the Michaelis-Menten curve fit, we calculated the K_m_ and V_max_ value of rEg-DHFR and human DHFR. The K_m_ and V_max_ value of rEg-DHFR were 18.74 ± 0.5651 μΜ and 2.384 ± 0.01874 μmol/min/mg, respectively; the K_m_ and V_max_ value of human DHFR were 14.61 ± 0.6590 μΜ and 3.370 ± 0.02327 μmol/min/mg, respectively. Methotrexate, aminopterin, trimethoprim and pyrimethamine were inhibitors of DHFR, which share structural similarities with the substrate of DHFR (DHF). The effect of inhibitors on the enzymatic activity were examined. Methotrexate and aminopterin were potent inhibitor with an IC_50_ of 27.75 ± 1.03 nM and 63.67 ± 6.76 nM for rEg-DHFR respectively, while pyrimethamine and trimethoprim showed lower inhibition effect with IC_50_ of 0.86 ± 0.0935 μM and 30.81 ± 2.09 μM, respectively (Fig. 5B–E and Table 1).

**Table 1:** IC_50_ values for inhibition of rEg-DHFR and human dihydrofolate reductase (DHFR) by various antifolates.

Antifolates	IC_50_ for rEg-DHFR	IC_50_ for human DHFR
Methotrexate	27.75 ± 1.03 nM	4.734 ± 0.569 nM
Aminopterin	63.67 ± 6.76 nM	253 ± 16.3 nM
Pyrimethamine	0.86 ± 0.0935 μM	4.26 ± 0.414 μM
Trimethoprim	30.81 ± 2.09 μM	127 ± 9.15 μM

*K*_*m*_ and *V*_*max*_ were determined for the substrate (DHF) in triplicate for rEg-DHFR and human DHFR. The data are expressed as the mean ± standard deviation (SD).

### The protoscolicidal activity of antifolates *in vitro*

**Figure 6:**
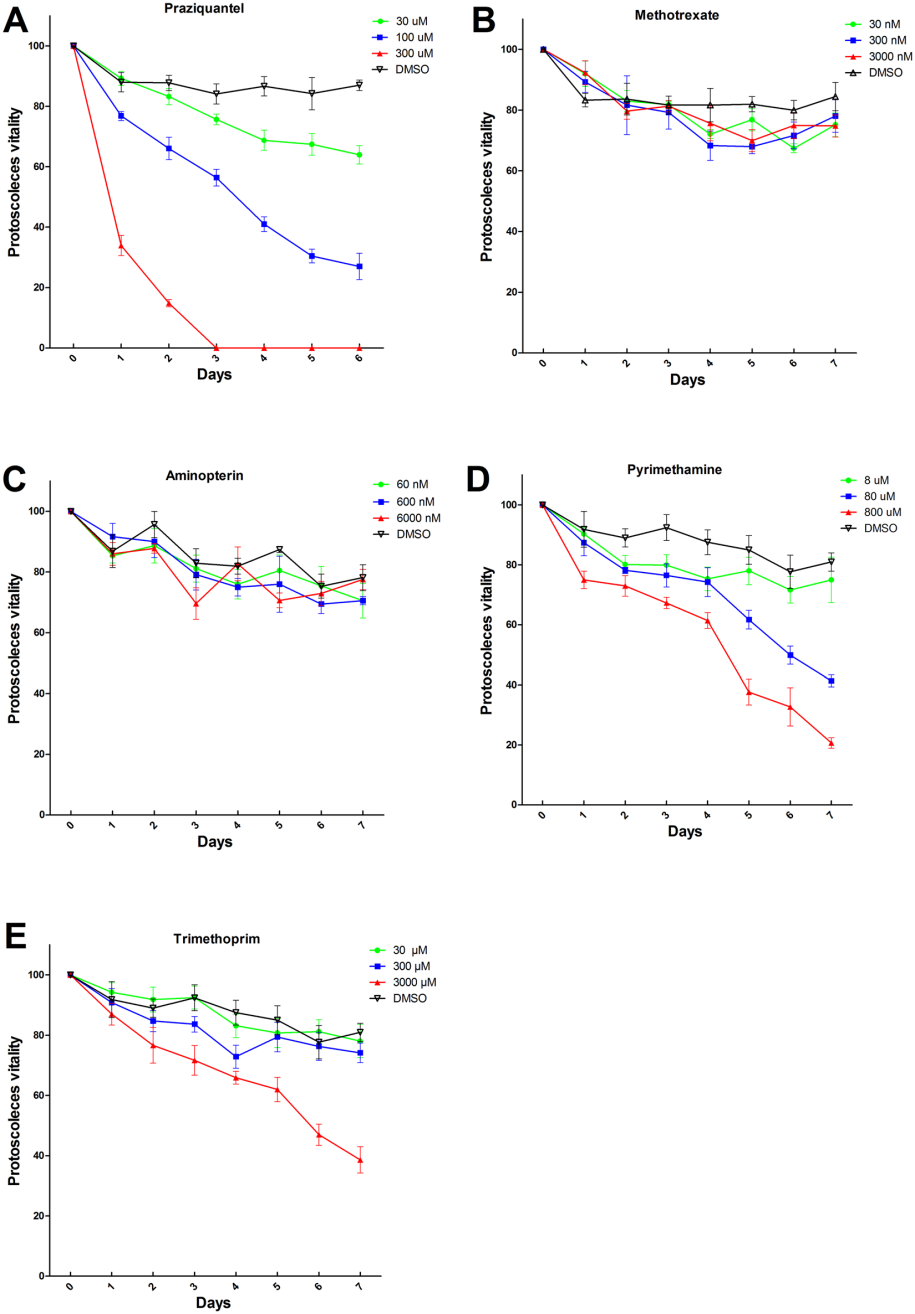
The protoscolicidal activity of antifolates *in vitro*. Vitality of protoscoleces (PSCs) following *in vitro* treatment with different concentrations of antifolates. Statistically significant differences were compared by analysis of variance (ANOVA). A value of P < 0.05 was considered statistically significant. (**A**) Praziquantel, ANOVA: F _(3, 24)_ = 10.87, P < 0.0001. (**B**) Methotrexate, ANOVA: F _(3, 28)_ = 0.3907, P = 0.7606. (**C**) Aminopterin, ANOVA: F _(3, 28)_ = 0.4456, P = 0.7223. (**D**) Pyrimethamine, ANOVA: F _(3, 28)_ = 4.431, P = 0.0114. (**E**) Trimethoprim, ANOVA: F _(3, 28)_ = 4.444, P = 0.113. Data shown are mean values from three experiments.

Based on the IC_50_ concentrations of antifolates derived from the enzyme activity inhibition assays, we treated the PSCs with these antifolates at different concentrations. Praziquantel was demonstrated to exhibit protoscolicidal activity, which was used as a positive control^30^. The survival of PSCs after exposure to antifolates were shown in Fig. 6. Control PSCs were not altered and remained viable (82.65 ± 6.11%) after 7 days of incubation. At all three concentrations, methotrexate and aminopterin showed slight effect on PSCs after 7 days of incubation. In the group treated with the highest concentration (800 μM) of pyrimethamine, the percentage vitality of PSCs was only 20.67 ± 2.49%. Trimethoprim showed a weaker protoscolicidal effect than pyrimethamine, with 38.67 ± 6.18% (3000 μM) of PSCs remaining viable in culture after 7 days.

### Establishment of indirect ELISA

Based on the checkerboard titration protocol, the optimal concentration of antigens and serum dilution were determined (Table 2). To determine the cut-off value of the indirect ELISA, 24 negative serum samples from sheep were tested in the optimised conditions. The mean OD_450_ value of rEg-DFHR ELISA was 0.255 and the standard deviation was 0.0488. Thus, the cut-off value was 0.4014 (mean + 3 SD). The cut-off values of other antigen ELISAs were listed in Table 2.

**Table 2:** Comparative evaluation of the serological assays for diagnosis of CE in sheep.

ELISAs	Optimal concentration of antigens	Optimal serum dilution	Cut-off values	Analytical specificity	Analytical sensitivity	Diagnostic specificity (DSP)	Diagnostic sensitivity (DSN)	Diagnostic accuracy
DHFR	0.6 μg/well	1:320	0.4014	85.7% (12/14)	1:6400	89.58% (43/48)	95.83% (23/24)	91.67% (66/72)
Tetraspanin-1	0.4 μg/well	1:640	0.6132	14.29% (2/14)	1:3200	43.75% (21/48)	75% (18/24)	54.17% (39/72)
Prohibitin	1.2 μg/well	1:80	0.6397	57.14% (8/14)	1:1600	66.67% (32/48)	50% (12/24)	61.11% (44/72)
Annexin	0.4 μg/well	1:640	0.5369	21.43% (3/14)	1:6400	83.33% (40/48)	87.5% (21/24)	84.72% (61/72)
Cystatin	0.8 μg/well	1:320	0.427	50% (7/14)	1:1600	77.08% (37/48)	79.17% (19/24)	77.78% (56/72)
Glutaredoxin-1*	1.6 μg/well	1:320	0.481	64.3% (9/14)	1:3200	100% (24/24)	95.83% (23/24)	97.9% (47/48)
Hydatid fluid	1:1600	1:160	0.467	71.43% (10/14)	1:1600	81.25% (39/48)	66.67% (16/24)	76.39% (55/72)

*Our previous published data, ref. 35.

Assessing the reproducibility and repeatability of our iELISA method, the interassay CVs ranged from 0.914% to 1.558% (mean = 1.153%), while the intra-assay CVs ranged from 0.583% to 2.014% (mean = 1.191%). The coefficients were <10%, which means this assay was repeatable and reproducible.

### Analytical sensitivity and specificity of the indirect ELISA

**Figure 7:**
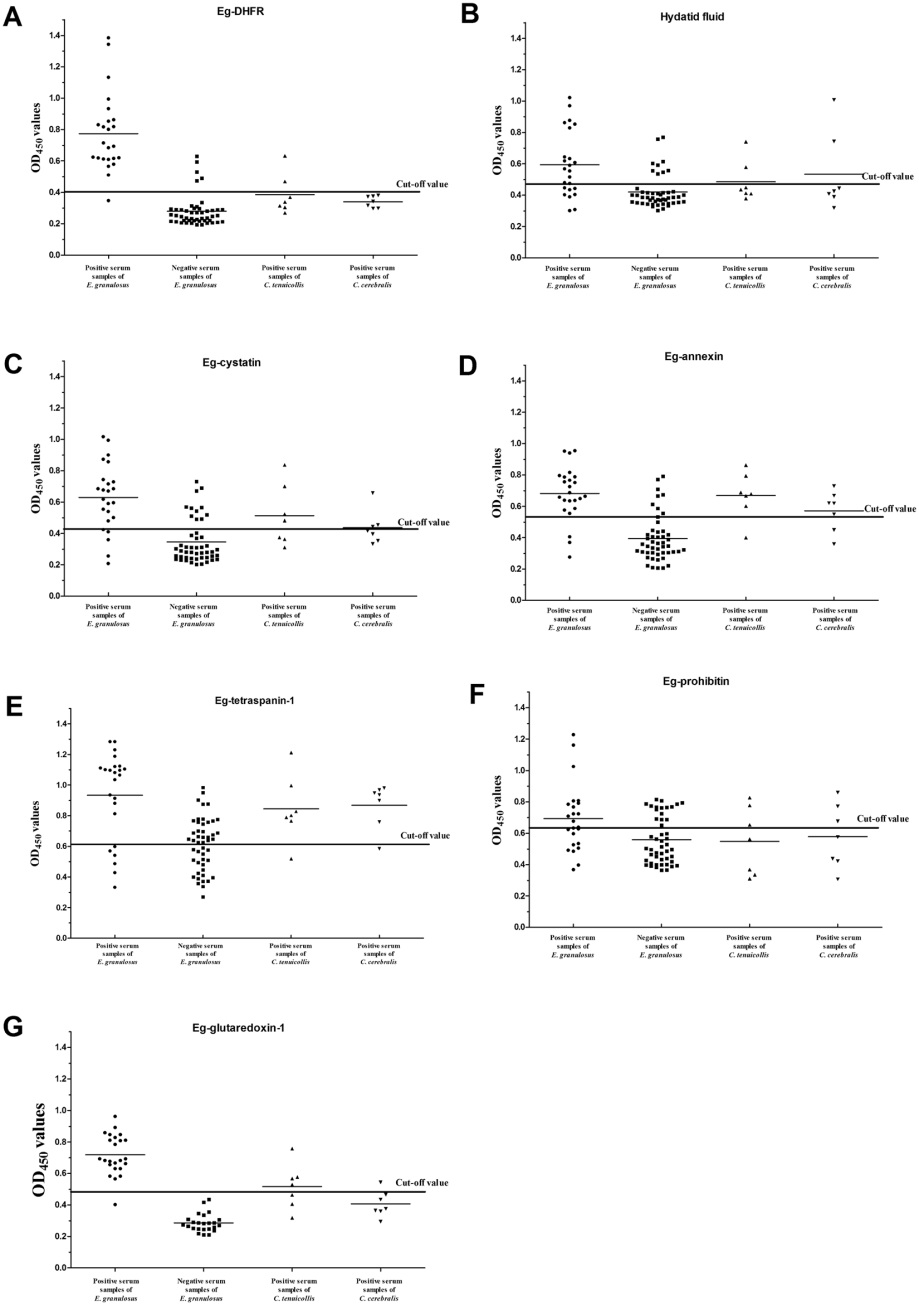
Indirect ELISAs (iELISAs) for the detection of cystic echinococcosis in sheep. (**A**) The rEg-DHFR iELISA. Statistically significant differences between *E. granulosus*-positive serum and the other positive sera were tested by one-way ANOVA using GraphPad Prism software (ANOVA: F _(2, 35)_ = 17.21, P < 0.0001). A statistically significant difference between the *E. granulosus*-positive group and the negative group was determined by *t*-test (*t-*test: t (70) = 12.04, P < 0.0001). P values < 0.05 were considered significant. (**B**) The hydatid fluid iELISA. ANOVA: F _(2, 35)_ = 0.8212, P = 0.4482; *t-*test: t (70) = 4.708, P < 0.0001. (**C**) The rEg-cystatin iELISA. ANOVA: F _(2, 35)_ = 3.019, P = 0.0617; *t-*test: t (70) = 6.765, P < 0.0001. (**D**) The rEg-annexin iELISA. ANOVA: F _(2, 35)_ = 1.324, P = 0.2791; *t-*test: t (70) = 7.411, P < 0.0001. (**E**) The rEg-tetraspanin-1 iELISA. ANOVA: F _(2, 35)_ = 0.4218, P = 0.6592; *t-*test: t (70) = 5.894, P < 0.0001. (**F**) The rEg-prohibitin iELISA. ANOVA: F _(2, 35)_ = 1.684, P = 0.2003; *t-*test: t (70) = 3.078, P = 0.003. (**G**) The rEg- glutaredoxin-1 iELISA (Our previous published data, ref. 35.). ANOVA: F (2, 18) = 28.41, P < 0.0001; *t-*test: t (46) = 15.12, P < 0.0001.

Positive serum samples from *Cysticercus tenuicollis*-infected goats and *Coenurus cerebralis*-infected sheep were tested to evaluate the cross-reactivity of this iELISA. Two *C. tenuicollis*-positive serum samples (n = 7) and no *C. cerebralis*-infected serum samples (n = 7) cross-reacted with Eg-DHFR (Fig. 7A). Thus, the analytical specificity of the rEg-DFHR ELISA was 85.7% (12/14). The analytical specificity of rEg-cystatin^31^, rEg-annexin^32^, rEg-tetraspanin-1^33^, rEg-prohibitin^34^, rEg-glutaredoxin-1^35^ and hydatid fluid were 50%, 21.43%, 14.29%, 57.14%, 64.3% and 71.43%, respectively (Table 2 and Fig. 7B–G). Compared with the cut-off value of rEg-DHFR ELISA (0.4014), the minimum detection (dilution) limit in serum was 1:6400 (mean absorbance = 0.527). The sensitivity of other antigens were shown in Table 2.

### Estimates of the indirect ELISA

Seventy-two sheep serum samples were used to analyze the diagnostic sensitivity (DSN), diagnostic specificity (DSP) and diagnostic accuracy of the ELISA. The assay of rEg-DFHR ELISA successfully confirmed infection in 23 of the infected sheep (23/24); the remainder were diagnosed as negative (Fig. 7). Thus, the DSN, DSP and diagnostic accuracy were 95.83% (23/24), 89.58% (43/48) and 91.67% (66/72) compared with the results of necropsy. The DSN, DSP and diagnostic accuracy of rEg-cystatin, rEg-annexin, rEg-tetraspanin-1, rEg-prohibitin and hydatid fluid ELISAs were lower than those of rEg-DFHR ELISA (Fig. 7 and Table 2).

## Discussion

DHFR catalyses the NADPH-dependent reduction of DHF to THF, which is involved in subsequent reactions such as thymidylate and purine nucleotide biosynthesis^36^. DHFR has been studied extensively in a variety of organisms, including mammals^37^, protozoa^38^, bacteria^39^ and several insects^27, 40^. Moreover, DHFR can be used as a drug target in the treatment of cancer and bacterial and parasitic infections^25^. However, the DHFR gene has not been described in tapeworms. This study characterised *E. granulosus* DHFR, and established an iELISA method based on the recombinant protein to diagnose CE in sheep.

The potential for using Eg-DHFR as a drug target for the treatment of *E. granulosus* has been analysed in our study. A comparison of the active site residues was performed between the DHFR sequences of mammalian hosts and *E. granulosus*, and they showed a difference of 45.7% (16/35). Among these different active site residues was the “REDMTFFS” motif, the region with the largest number of active site residues, which contained many potentially significant changes with respect to the design of non-mammalian specific DHFR inhibitors. As observed in previous studies of non-mammalian DHFRs, there were also residue charge changes at the NADPH binding site between *H. sapiens* DHFR (negative charge) and Eg-DHFR (positive charge), which results in a different direction and size of the loop on the NADPH site of the two enzymes. This charge and conformation change raise the possibility of designing specific inhibitors directed towards *E. granulosus* by introducing a negatively charged group at this position^27, 29^. Simultaneously, it is interesting to note that modelled three-dimensional structure changes between mammalian hosts and *E. granulosus* DHFR at two positions, where the α-helix is replaced as random coil (Asn64 - Phe64) or the random coil is replaced as α-helix (Trp24 - Trp26) (Fig. 2A). These differences in both residue type and three-dimensional structure lead to the appearance of a deep, narrow groove in the surface of *E. granulosus* DHFR, but not in the surface of human DHFR. This is a very attractive feature as its great significance for the design of inhibitors with high selectivity for *E. granulosus* DHFR. In addition, other potential significant differences are in the glutamate sub-site and the p-aminobenzamide binding region, where residues Met32 and Phe64 in *E. granulosus* are changed to be Phe31 and Asn64 (Fig. 2B). Referring to previous studies, this could expose a larger hydrophobic binding pocket in Eg-DHFR and provide a significant reference for the design of inhibitors^27, 29^.

Recombinant Eg-DHFR exhibited enzymatic parameters towards the substrate DHF similar to those of other DHFRs (K_m_ of 18.74 ± 0.5651 μM and V_max_ of 2.384 ± 0.01874 μmol/min/mg)^40, 41^. Native Eg-DHFR is difficult and expensive to prepare. However, rEg-DHFR retains enzymatic activity that can be used to further screen newly designed drugs to inhibit *E. granulosus*, which greatly reduces the difficulty of the screening of inhibitors. The inhibitors of DHFR belong to antifolates, which share structural similarities with the substrate of DHFR (DHF). The effect of antifolates on the enzymatic activity of rEg-DHFR were investigated in this study. Methotrexate and aminopterin were shown to be stronger inhibitors of both rEg-DHFR and human DHFR enzymes than pyrimethamine and trimethoprim. It is attributed to the fact that methotrexate and aminopterin have a pteridine ring and glutamic acid, which are the most similar to the normal substrate of DHFR (DHF), and lead to the effective inhibition (Fig. 5). Due to the inhibitory effect of on the activity of the rEg-DHFR enzyme, it is interesting to explore whether these antifolates could affect PSCs *in vitro*. Consequently, even at concentrations that are orders of magnitude higher than the IC_50_ for EgDHFR, there is little effect on protoscolex viability. Therefore, although the antifolates were very effective against EgDHFR, inhibition of DHFR is of little consequence to protoscolex viability *in vitro*.

There were no research on the location of DHFR in parasitic helminths. Our results revealed that Eg-DHFR was widely distributed in the larva, germinal layer, and adult worm of *E. granulosus*. In particular, a high level of Eg-DHFR protein was detected in the rostellum and suckers (scolex) of adult worms, but not in PSCs. The rostellum and suckers of adult worms are involved in absorption of nutrition and adhesion to the walls of the small intestine of the definitive host, which are of major importance for the survival of the parasite^42, 43^.

CE is a worldwide economic and public health problem. Currently, detection and surveillance of *E. granulosus* infection in livestock relies on necropsy and macroscopic observation procedures in abattoirs^44, 45^, and these detections without histological examination have a high error rate (15.4%)^46^. It is important to establish an inexpensive, accurate immunodiagnostic assay as a monitoring tool for the detection of CE in live animals^17^. The antigen B (8 kDa) from hydatid cyst fluid has high diagnostic sensitivity and specificity (over 90%) to detect CE in sheep^18, 19^. However, it is hard to purify and standardise large amounts of natural *E. granulosus* hydatid cyst fluid antigens. Thus, recombinant protein antigens have become an important way to enhance immunodiagnosis of CE in sheep. Previous research using the recombinant EG95 oncosphere protein and recombinant AgB protein as antigens in ELISA exhibited poor diagnostic sensitivity, 1.6% and 28% for sheep CE, respectively^15, 21^. Here, an iELISA diagnostic method based on recombinant Eg-DHFR was successfully established and optimised to detect *E*. *granulosus* infection in sheep. Compared with iELISAs using other recombinant antigens of *E. granulosus* (rEg-cystatin, rEg-annexin, rEg-tetraspanin-1, rEg-prohibitin and hydatid fluid), recombinant Eg-DHFR had high analytical sensitivity (1:6400) and analytical specificity (85.7%). There was no cross-reaction detected with *C. cerebralis*-positive serum. However, other antigens showed three to seven cross reactions with *C. cerebralis*-positive serum. DSN, DSP and diagnostic accuracy were 95.83% (23/24), 89.58% (43/48) and 91.67% (66/72) compared with the results of necropsy, which suggested that the recombinant Eg-DHFR could clearly react with *E. granulosus*-specific IgG antibodies. Recombinant Eg-DHFR had a similar diagnostic performance as rEg-glutaredoxin-1 compared with our previous report^35^. Although a lower cross-reaction ratio of rEg-DHFR was observed when against taeniid cestodes positive sera compared with rEg-glutaredoxin-1. However, the difference is not significant because of the small sample size. Therefore, the cross-reaction ratio should be calculated more accurate by further study using large sample number.

## Conclusion

We identified a new *E*. *granulosus* diagnostic antigen candidate distributed in all life-cycle stages of the parasite. The bioinformatic, tissue distributions and enzymatic characteristics of Eg-DHFR was made in this study. The results demonstrate that Eg-DHFR is a potential drug target for control of the important cestode parasite *E*. *granulosus*. Furthermore, we established an iELISA based on rEg-DHFR, which might be a promising tool for diagnosis and serosurveillance of *E. granulosus* infections in sheep.

## Methods

### Parasites and animals

Cysts of *E. granulosus* were obtained from a slaughterhouse in Qinghai Province, China. The PSCs were separated in sterile conditions and washed three times in phosphate-buffered saline (PBS). Rinsed PSCs were diluted to a concentration of 1,500 mL^−1^ and maintained as previously described^33, 47^. Briefly, PSCs were cultured in RPMI 1640 medium (Hyclone) with 10% fetal bovine serum (Hyclone), 10% hydatid fluid, 100 U.mL^−1^ penicillin G and 100 μg.mL^−1^ streptomycin (Sigma, USA). Adult worms were collected from the small intestine of a 2-month-old dog 35 days post-infection with 20,000 PSCs. A 5-month-old dog and two 9-week-old female New Zealand white rabbits were obtained from the Laboratory Animal Center of Sichuan Agricultural University. All animals were provided with food pellets and sterilised water ad libitum.

### Ethics statement

All animals were handled in strict accordance with the animal protection law of the People’s Republic of China (a draft animal protection law was released on September 18, 2009). All procedures were performed in accordance with the rules of the Care and Use of Laboratory Animals of the Animal Ethics Committee of Sichuan Agricultural University (Ya’an, China) (Approval No. 2013–028). All the methods were carried out in accordance with relevant guidelines and regulations, including any relevant details.

### Bioinformatic analysis of Eg-DHFR coding sequence

The full-length Eg-DHFR sequence was downloaded from GeneDB (EgrG_000572400) (http://www.genedb.org/Homepage). The ExPASy Proteomics Server (http://expasy.org/) was used to predict conserved domains and the molecular weight of Eg-DHFR. ClustalX software version 1.83 was used for multiple sequence alignment^33^. Three-dimensional structural modelling was performed using the SWISS-MODEL server (http://swissmodel.expasy.org) based on the crystal structure of *Schistosoma mansoni* DHFR (PDB accession code: 3vco.1), which has a resolution of 1.95 Å (to be published). The three-dimensional structure of Eg-DHFR was compared with those of the homologous proteins from *H. sapiens* and *S. mansoni*. The crystal structure of *H. sapiens* DHFR (PDB accession code: 3gyf.1) has a resolution of 1.70 Å^48^.

### Expression and purification of rEg-DHFR

The region encoding mature Eg-DHFR was amplified from *E. granulosus* cDNA using the primers 5′-CGCGGATCCATGGGGCTGAAGCGTCT-3′ and 5′-CCGCTCGAGATGATCATTAAGGGGATGCG-3′, and then ligated into the *Bam*HI/*Xho*I restriction sites of vector pET-28a(+) (Novagen, Madison, WI, USA). Recombinant protein was expressed and purified as previously described^33^. Briefly, the recombinant plasmid was transformed into *E. coli* BL21 (DE3) cells (Invitrogen, Carlsbad, CA, USA) and expression was induced with 1 mM isopropyl- thio-β-D-1-thiogalactopyranoside at 37 °C for 5 h. The bacterial cells were harvested and suspended in lysis buffer, followed by ultrasonic lysis. The recombinant protein was purified by Ni^2+^ affinity chromatography (Bio-Rad, Hercules, CA, USA) following the manufacturer’s instructions. Recombinant protein was detected by 12% SDS-PAGE and the concentration of protein was estimated using a bicinchoninic acid protein assay kit (Pierce, Rockford, IL, USA).

### Serum and antigen preparation

Positive serum against *E. granulosus* (24 samples) was collected from naturally infected sheep in Ganzi Tibetan Autonomous Prefecture, Sichuan Province, China. Goat serum positive against C*ysticercus tenuicollis* (7 samples) and sheep serum positive against *Coenurus cerebralis* (7 samples) was also obtained from farms in Sichuan Province. Negative serum (72 samples) was collected from 72 healthy sheep with no cysts (determined by autopsy) from Xichang, Sichuan Province. The polyclonal antibody against rEg-DHFR was obtained as previously described^33^. Briefly, each rabbit was inoculated subcutaneously with 200 μg rEg-DHFR emulsified in Freund’s complete adjuvant (Sigma, St. Louis, MO, USA), followed by three repeat inoculations every 2 weeks with 200 μg rEg-DHFR emulsified in Freund’s incomplete adjuvant (Sigma). Rabbit anti-rEg-DHFR serum was detected by ELISA and purified using HiTrap Protein A affinity chromatography (Bio-Rad). The rEg-cystatin, rEg-annexin, rEg-tetraspanin-1 rEg-prohibitin and rEg-glutaredoxin-1 (the gene accession number are EgrG_000849600, EgrG_000041300, FJ384717, KT149769 and EgrG_000124800, respectively) were all produced similarly as described above. The crude hydatid fluid antigen was extracted from the hydatid cysts with 20 ml syringe. Then the fluid was clarified by centrifugation at 10,000 rpm for 20 min and filtered through a 0.22 μm membrane^19, 49^.

### Immunoblot and immunohistochemical analyses

The total proteins of PSCs were obtained with a mammalian protein extraction kit (CWBIO, Beijing, China). Purified rEg-DHFR and total PSC proteins were separated by 12% SDS-PAGE and transferred onto nitrocellulose membranes. The membranes were blocked with 5% (w/v) skim milk for 2 h at room temperature. Subsequently, the membranes were incubated with *E. granulosus*-infected sheep serum or anti-rEg-DHFR rabbit IgG (1:200 v/v dilutions) overnight at 4 °C. Following washing, the membranes were incubated with horseradish peroxidase (HRP)-conjugated goat anti-rabbit IgG or rabbit anti-sheep IgG (1:3,000 dilutions; Boster Bio-project Co, Wuhan, China) for 1 h, respectively. Signals were visualised using an Enhanced HRP-DAB Chromogenic Substrate Kit (Tiangen, Beijing, China). Negative controls were performed with serum of healthy sheep and preimmune rabbit.

Immunolocalisation studies were performed as previously described with some modifications^33^. Briefly, fresh adult worms, PSCs and germinal layer were fixed and embedded in paraffin. The sections were prepared with 0.01 M citrate buffer (pH 6.0) for 12 min for heat-induced antigen retrieval. Then the sections were probed with purified rabbit anti-rEg-DHFR IgG (1:200 dilution) and reacted with fluorescein (FITC)-conjugated goat anti-rabbit IgG (H+L) (1:200 dilution in 1% Evans Blue; Bethyl Laboratories, Montgomery, TX, USA) for 1 h at 37 °C in darkness. The nucleus was stained with 4′,6-diamidino-2-phenylindole (DAPI; Sigma, USA) for 5 min. The sections were imaged with a fluorescence microscope (Nikon, Tokyo, Japan). Negative controls were performed with preimmune rabbit serum.

### Enzyme assay

The enzymatic characteristics of rEg-DHFR and human DHFR enzyme (DHFR assay kit; Sigma) were analysed using a DHFR assay kit (Sigma) following the manufacturer’s instructions^41^. Briefly, a range of purified rEg-DHFR concentrations was added to the assay buffer and mixed well and the optimal concentration was determined. Reaction progress was measured based on the decrease in absorbance at 340 nm. Data was recorded at 25 s intervals for 2.5 min. The temperature was controlled at 22 °C. The extinction coefficient for the DHFR reaction was 12.3 mM^−1^ cm^−1^ at 340 nm. One unit was defined as the amount of enzyme required to convert 1 μmol of DHF per min to THF at 22 °C. Thereafter, to estimate the kinetic parameters maximal velocity (V_max_) and Michaelis constant (K_m_), different concentrations (ranging from 0 μM to 120 μM) of DHF and a constant concentration (60 μM) of NADPH were added into the reaction system. Kinetic parameters (V_max_ and K_m_) were calculated using GraphPad Prism® software (San Diego, CA, USA; http://www.graphpad.com) based on a Michaelis-Menten curve fit.

### Determination of IC_50_ for antifolates

Different concentrations of antifolates (Sigma) were used to measure the 50% inhibition of the enzyme reaction (IC_50_) containing 50 μM dihydrofolic acid and 60 μM NADPH. The IC_50_ was calculated using GraphPad Prism software by nonlinear curve-fitting.

### Antifolates treatment of *E. granulosus* protoscoleces

PSCs were cultured in 24-well microplates (1,500 PSCs/well in 1 ml medium) and incubated with different concentrations of praziquantel (30 μM, 100 μM and 300 μM,), methotrexate (30 nM, 300 nM and 3000 nM), aminopterin (60 nM, 600 nM and 6000 nM), pyrimethamine (8 μM, 80 μM and 800 μM) and trimethoprim (30 μM, 300 μM, 3000 μM). PSCs incubated with only assay buffer (DHFR assay kit; Sigma) or DMSO were used as negative controls. The vitality of protoscoleces was assessed using a Trypan Blue exclusion test^50, 51^. The daily percentages of PSCs vitality were calculated on six microscopic fields covering approximately 10–20 PSCs. The effect of per drug concentration was monitored in triplicate and in two separate trials.

### Establishment of the indirect ELISA

ELISAs were performed essentially as described^52, 53^. Briefly, the optimal concentration of antigens (rEg-DHFR, rEg-cystatin, rEg-annexin, rEg-tetraspanin-1, rEg-prohibitin and hydatid fluid antigens) and serum were assessed by standard checkerboard titration procedures. The ELISA plates were coated with six different concentrations overnight at 4 °C. After washing, the plates were blocked with 5% skim milk for 1.5 h at 37 °C. After the blocking solution was discarded, the plates were incubated with sheep serum samples in twofold dilutions ranging from 1:20 to 1:2560. Subsequently, the plates were incubated with a 1:200 dilution of HRP-conjugated rabbit anti-goat/sheep IgG (Boster Bio-project Co) at 37 °C- for 1 h. Following washing, antibody binding was detected with 100 μL of 3, 3′, 5, 5′-tetramethylbenzidine (Tiangen). After stopping the reaction, the absorbance was determined at 450 nm in a microplate reader (Thermo Scientific, Pittsburgh, PA, USA). An OD_450_ value for positive serum close to 1.0 and the highest P/N value between positive and negative serum were regarded as optimal. The cut-off value was calculated as the mean OD_450_ absorbance value for the 24 negative serum samples plus three standard deviations (3 SD). Intraplate repeatability was evaluated by the coefficient of variation (CV %) of every serum sample. Three separate assays were used to evaluate the intraplate repeatability.

### Analytical sensitivity and specificity of indirect ELISA

To evaluate the analytical sensitivity of the indirect ELISA, three *E. granulosus*-positive sheep serum samples were diluted in twofold series from 1:100 to 1:102,400. The minimum detection limit of the positive serum samples was determined as the analytical sensitivity of the indirect ELISA by comparing with the cut-off value^54^. To evaluate the analytical specificity of the indirect ELISA, cross-reactions were tested with *C. cerebralis*-positive serum and *C. tenuicollis*-positive serum. The percentage analytical specificity was calculated as indirect ELISA negative × 100/true negative.

### Estimates of the indirect ELISA

The DSN, DSP and diagnostic accuracy of the indirect ELISA was evaluated using 72 sheep serum samples, including 24 positive sera and 48 negative sera. The DSN, DSP and accuracy of the iELISA were calculated based on the following formulae^54^: DSN = true-positive/(true-positive + false-negative) × 100, DSP = true-negative/(true-negative + false-positive) × 100 and diagnostic accuracy = (true-positive + true-negative)/total number × 100. Each serum sample was tested three times.

### Statistical analysis

All data are presented as the mean ± SD. Statistical analyses were performed by *t*-test and one-way ANOVA for comparison between groups using the software package GraphPad Prism. P values < 0.05 were considered significant.

## Electronic supplementary material

Dataset 1
