# Supplementary material for: Molecular Characteristics and Serodiagnostic Potential of Dihydrofolate Reductase from Echinococcus granulosus
Source: Sci Rep. 2017 Mar 31;7:514. doi: 10.1038/s41598-017-00643-5 (PMC5428777; doi:10.1038/s41598-017-00643-5)
Supplement: Supplementary file 1 — Dataset 1 [file 41598_2017_643_MOESM1_ESM.pdf]

Supplementary Information

Molecular Characteristics and Serodiagnostic Potential of Dihydrofolate Reductase from *Echinococcus granulosus*

Xingju Song[1¶], Dandan Hu[1¶], Min Yan[1], Yu Wang[1], Ning Wang[1], Xiaobin Gu[1], Guangyou Yang[1 *]

[1] Department of Parasitology, College of Veterinary Medicine, Sichuan Agricultural University, Chengdu, China

*Corresponding author

E-mail: guangyou1963@aliyun.com

[¶] These authors contributed equally to this work.

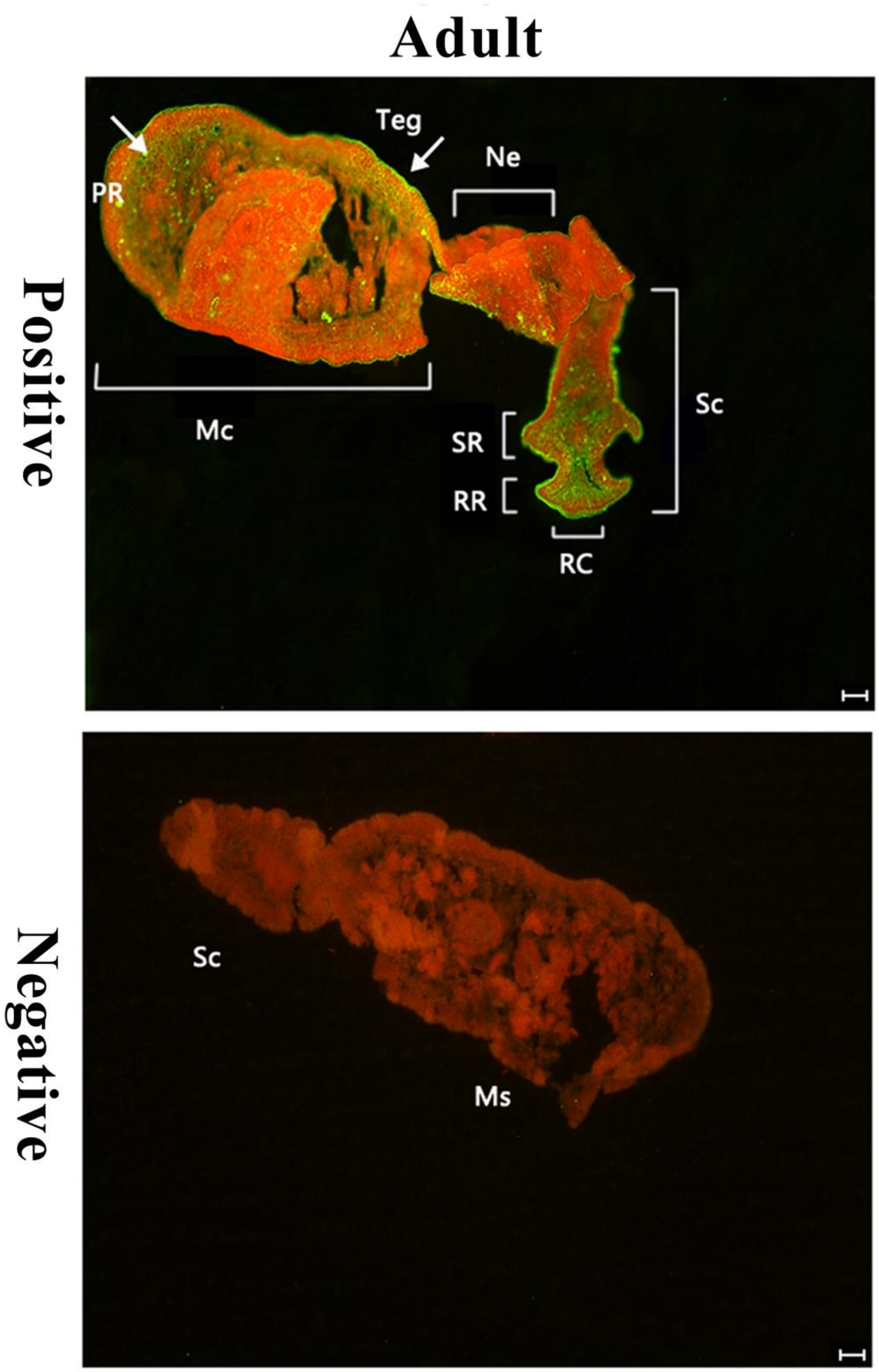

Figure S1. Immunofluorescent localisation of Eg-DHFR in adult worm.

Abbreviations: RR, rostellum region; SR, sucker region; RC, rostellar cone; Sc, scolex; Ne, neck; Ms, mature segment. Scale bars: 50 µm.
